# Anti-inflammatory effects of BHBA in both *in vivo* and *in vitro* Parkinson’s disease models are mediated by GPR109A-dependent mechanisms

**DOI:** 10.1186/s12974-014-0230-3

**Published:** 2015-01-17

**Authors:** Shou-Peng Fu, Jian-Fa Wang, Wen-Jing Xue, Hong-Mei Liu, Bing-run Liu, Ya-Long Zeng, Su-Nan Li, Bing-Xu Huang, Qing-Kang Lv, Wei Wang, Ju-Xiong Liu

**Affiliations:** College of Veterinary Medicine Jilin University, Changchun, 130062 P R China; College of Animal Science and Veterinary Medicine, Heilongjiang Bayi Agricultural University, Daqing, 163319 P R China

**Keywords:** BHBA, GPR109A, Parkinson’s disease, neuroinflammation, LPS, NF-κB

## Abstract

**Background:**

Accumulating evidence suggests that neuroinflammation plays an important role in the progression of Parkinson’s disease (PD). Excessively activated microglia produce several pro-inflammatory enzymes and pro-inflammatory cytokines, leading to damage to surrounding neurons and eventually inducing neurodegeneration. Therefore, the inhibition of microglial overactivation may be a potential therapeutic strategy to prevent the further progression of PD. β-Hydroxybutyric acid (BHBA) has been shown to suppress lipopolysaccharide (LPS)-induced inflammation in BV-2 cells and to protect dopaminergic neurons in previous studies, but the underlying mechanisms remain unclear. Thus, in this study, we further investigated this mechanism in LPS-induced *in vivo* and *in vitro* PD models.

**Methods:**

For the *in vitro* experiments, primary mesencephalic neuron-glia cultures were pretreated with BHBA and stimulated with LPS. [^3^H]dopamine (DA) uptake, tyrosine hydroxylase-immunoreactive (TH-ir) neurons and morphological analysis were evaluated and analyzed in primary mesencephalic neuron-glia cultures. *In vivo*, microglial activation and the injury of dopaminergic neurons were induced by LPS intranigral injection, and the effects of BHBA treatment on microglial activation and the survival ratio and function of dopaminergic neurons were investigated. Four our *in vitro* mechanistic experiment, primary microglial cells were pretreated with BHBA and stimulated with LPS; the cells were then assessed for the responses of pro-inflammatory enzymes and pro-inflammatory cytokines, and the NF-κB signaling pathway was evaluated and analyzed.

**Results:**

We found that BHBA concentration-dependently attenuated the LPS-induced decrease in [^3^H]DA uptake and loss of TH-ir neurons in the primary mesencephalic neuron/glia mixed culture. BHBA treatment significantly improved the motor dysfunction of the PD model rats induced by intranigral injection of LPS, and this beneficial effect of BHBA was attributed to the inhibition of microglial overactivation and the protection of dopaminergic neurons in the substantia nigra (SN). Our *in vitro* mechanistic study revealed that the inhibitory effect of BHBA on microglia was mediated by G-protein-coupled receptor 109A (GPR109A) and involved the NF-κB signaling pathway, causing the inhibition of pro-inflammatory enzyme (iNOS and COX-2) and pro-inflammatory cytokine (TNF-α, IL-1β, and IL-6) production.

**Conclusions:**

In conclusion, the present study supports the effectiveness of BHBA in protecting dopaminergic neurons against inflammatory challenge.

## Background

Parkinson’s disease (PD) is the second most prevalent neurodegenerative disorder, affecting millions of people worldwide [1]. A major hallmark of PD is the loss of dopaminergic neurons in the substantia nigra par compacta (SNpc) of the midbrain [2]. The loss of dopaminergic neurons in PD leads to motor dysfunction accompanied by progressive non-motor symptoms, which include cognitive impairments, mood disturbances, sleep dysfunction, gastrointestinal problems, and dysautonomia [3-5]. Although the exact mechanisms underlying PD pathogenesis are yet to be defined, oxidative stress, mitochondrial dysfunction, and inflammation may contribute to this process [6-8].

Accumulating evidence suggests that neuroinflammation plays an important role in the progression of PD [9,10]. Post-mortem studies have shown that there is a large number of reactive microglia in the substantia nigra (SN) in PD, particularly in areas of maximal neurodegeneration, namely the ventral and lateral regions of the SN [11]. A robust activation of microglia has also been found in both 1-methyl-4-phenyl-1, 2, 3, 6-tetrahydropyridine (MPTP)- and 6-hydroxydopamine (6-OHDA)-induced PD animal models [12,13]. Uncontrolled overactivation of microglia is a major component of neuroinflammation. Excessive activation of microglia and the consequent release of several pro-inflammatory cytokines and/or pro-inflammatory enzymes, such as TNF-α, IL-1β, IL-6, inducible nitric oxide synthase (iNOS), and cyclooxygenase-2 (COX-2), are believed to contribute to neurodegenerative processes [14,15]. Therefore, inhibition of microglial overactivation may be a potential therapeutic strategy to prevent further progression of PD.

In mesencephalic neuron-glia cultures, the stimulation of microglia with inflammagen lipopolysaccharide (LPS) induces the production of factors, including TNF-α, IL-1β, IL-6, iNOS, and COX-2 [16,17]. Studies have attributed the accumulation of these factors to the degeneration of dopaminergic neurons [18-20]. The intranigral infusion of LPS in rats results in the significant degeneration of nigral dopaminergic neurons and depletion of striatal dopamine (DA) [21,22]. Therefore, these *in vitro* and *in vivo* models of inflammation-mediated dopaminergic neurodegeneration are powerful tools in mechanistic studies and the identification of potential therapeutic agents.

β-Hydroxybutyric acid (BHBA) is an important intermediate of amino and fatty acid catabolism that has been demonstrated to be neuroprotective [23,24]. Previous studies have shown that BHBA has strong protective effects in an MPTP-induced PD mouse model [25] and provides substantial protection against apoptosis of dopaminergic neurons intoxicated by 1-methyl-4-phenylpyri-dinium (MPP^+^) [24], demonstrating that it is a potent neuroprotectant in both *in vivo* and *in vitro* PD models. Previous mechanistic studies have revealed that the anti-inflammatory effects of BHBA contributed to its neuroprotective effects [15,26], but the precise underlying mechanism is still unclear. The purpose of the present study was to investigate the neuroprotective and anti-inflammatory properties of BHBA in LPS-induced *in vivo* and *in vitro* PD models and to identify the specific anti-inflammatory mechanism of BHBA.

## Methods

### Animals and surgery

Male Wistar rats (250 to 290 g) were obtained from the Center of Experimental Animals of the Baiqiuen Medical College of Jilin University (Jilin, China). The rats were maintained in plastic cages under conventional conditions. Water and pelleted diets were supplied *ad libitum*. Studies were performed in accordance with the guidelines established by the Jilin University Institutional Animal Care and Use Committee. The animals were allowed to acclimate to their new surroundings for 7 days before experimental manipulations. They were anesthetized with sodium pentobarbital (45 mg/kg, i.p.) and positioned in a stereotaxic apparatus (David Kopf Instruments, Tujunga, CA, U.S.A) to conform to the brain atlas of Paxinos and Watson [27]. LPS (obtained from *Escherichia coli*, serotype O26:B6; Sigma-Aldrich, St. Louis, MO, USA) were dissolved (5 mg/ml) in phosphate-buffed saline (PBS), and 2.0 μl was injected into the right SNpc at a rate of 0.2 μl/min. The injection needle was lowered through a drill hole at 5.3 mm posterior, 2 mm lateral, and 7.8 mm ventral to the bregma. The injections were delivered over a period of approximately 10 min. Then, the needle was left *in situ* for 5 min to avoid reflux along the injection track. Thereafter, the skull surface was covered with fibrosponge, and the skin was sutured. Sham-operated animals were subjected to the same surgical procedures, except that 2 μl of PBS was injected into the SNpc.

### Application of β-hydroxybutyric acid

Rats were divided into the following five groups: the sham-operated group, the LPS-injected group followed by vehicle treatment, and the LPS-injected group followed by treatment with 0.4, 0.8, or 1.6 mmol/kg/d BHBA (Sigma-Aldrich, St. Louis, MO, USA). BHBA was resolved in PBS and administered subcutaneously (1 μl/h) using Alzet mini-osmotic pumps (DURECT Corp., Cupertino, California, CA, USA). The rats received BHBA from 3 days before LPS injection up to 21 days post-LPS injection (24 days in total).

### Rotational behavior assay

A rotational behavior assay was performed according to a previously described protocol [21,22]. Briefly, rats were placed into cylinders attached to a rotameter (Columbus Instruments, Columbus, OH, USA) and allowed to adapt for 10 min to the testing environment. Then, they were intraperitoneally injected with 5 mg/kg D-amphetamine sulfate (Sigma-Aldrich, St. Louis, MO, USA) dissolved in physiological saline. Measurements of rotational activity began at 5 min after injection and lasted for 30 min under minimal external stimuli. The number of turns made during the entire 30-min testing period was counted.

### Rat mesencephalic neuron-glia cultures

Embryonic mesencephalic neuron-glia cultures were obtained from timed-pregnant Wistar rats on embryonic day 14. Briefly, ventral mesencephalic tissues were removed and dissociated to single cells by a mechano-enzymatic method involving a protease treatment with 2.5 mg/ml trypsin and 0.1 mg/ml DNAse type I (Sigma-Aldrich, St. Louis, MO, USA) and additional mechanical shearing. Cells were seeded at 2 × 10^5^ per well in 24-well culture plates precoated with poly-D-lysine (1 mg/ml) (Sigma-Aldrich, St. Louis, MO, USA) and maintained at 37°C in a humidified atmosphere of 5% CO_2_ and 95% air in a maintenance medium consisting of minimum essential medium supplemented with 10% heat-inactivated fetal bovine serum and 10% heat-inactivated horse serum (Gibco Life Technologies, Inc., Grand Island, NY), 2 mM L-glutamine, 1 mM sodium pyruvate, 100 μM nonessential amino acids, 50 U/ml penicillin, and 50 μg/ml streptomycin (Gibco Life Technologies, Inc., Grand Island, NY). Seven-day-old cultures were used for the treatment.

### Primary microglia-enriched cultures

Rat microglia-enriched cultures were prepared according to a previously described protocol [28,29]. Briefly, whole brains of 1-day-old neonatal Wistar rats, with the blood vessels and meninges removed, were triturated in Hank’s balanced salt solution. Cells (2.5 × 10^7^) were seeded in 150-cm^2^ culture flasks in 15 ml of a Dulbecco’s modified Eagle’s medium/nutrient mixture F12 mixture (1:1) (Gibco Life Technologies, Inc., Grand Island, NY) containing 10% heat-inactivated FBS, 2 mM L-glutamine, 1 mM sodium pyruvate, 100 μM nonessential amino acids, 50 U/ml penicillin, and 50 μg/ml streptomycin. The cultures were maintained at 37°C in a humidified atmosphere of 5% CO_2_ and 95% air. The medium (15 ml/flask) was replenished at 1 and 4 days after the initial seeding and changed every third day thereafter. Upon reaching confluence (day 14), the microglia were shaken off (200 rpm for 4 h on an orbital shaker), pelleted at 800 g for 10 min, resuspended in fresh medium, and plated (10^5^ cells/well) in 24-well culture plates. Twenty-four hours later, the cells were ready for treatment. The purity of the microglial culture was >98% as previously determined by immunofluorescence and cytochemical analysis [30].

### [^3^H]DA uptake assay

Cultures were incubated for 20 min at 37°C with 1 μM [^3^H]dopamine (DA) in Krebs-Ringer buffer (Sigma-Aldrich, St. Louis, MO, USA). After washing three times with ice-cold Krebs-Ringer buffer, the cells were lysed in 1 N NaOH. A liquid scintillation counter (Tri-Carb, model 3314, Packard) was used for measuring radioactivity. Nonspecific DA uptake observed in the presence of mazindol (10 μM) was subtracted.

### High-performance liquid chromatography

High-performance liquid chromatography (HPLC) analysis was performed according to a previously described protocol for DA and its metabolite 3,4-dihydroxyphenylacetic acid (DOPAC) [21,22]. Briefly, SNs were weighed and suspended in 200 mM ice-cold perchloric acid. Each sample was sonicated and then placed in an ice bath for 60 min. Subsequently, the sample was centrifuged at 20,000 g for 20 min at 4°C. The supernatant was transferred to a clean tube, and the volume was measured. One-half volume of a potassium dihydrogen phosphate solution was added to the supernatant and centrifuged at 20,000 g for 20 min at 4°C. An aliquot of the supernatant was injected into an HPLC system for analysis.

### RNA interference

G-protein-coupled receptor 109A (GPR109A) siRNAs were purchased from OriGene (OriGene Technologies, Beijing, China) and complexed with Lipofectamine 2000 (Invitrogen, Carlsbad, CA, USA) in 24-well plates, according to the manufacturer’s instructions.

### RNA extraction, reverse transcription and quantitative real-time PCR

Total RNA was extracted from the cells using Trizol (Invitrogen, Carlsbad, CA, USA), according to the supplier’s protocol. Total RNA was then treated with RNase-free Dnase I, quantified by measuring the absorbance at 260 and 280 nm and stored at −80°C until analysis. The extracted RNA was subjected to RT-PCR using a PrimeScript RT Reagent Kit With gDNA Eraser (Takara Shuzo Co., Ltd., Kyoto, Japan). The mRNA levels of various genes were evaluated by quantitative polymerase chain reaction (qRT-PCR) using a SYBR Green QuantiTect RT-PCR Kit (Roche, South San Francisco, CA, USA), and each sample was assessed in triplicate. The relative expression levels of iNOS, COX-2, TNF-α, IL-1β, IL-6, and GPR109A were calculated relative to β-actin (the normalizer) using the comparative cycle threshold method. The primer sequences for the tested genes are shown in Table 1.

**Table 1:** **The primer sequences of β-actin, GPR109A, iNOS, COX-2, TNF-α, IL-1β, and IL-6**

**Gene**	**Sequences**	**Length (bp)**
β-actin	(F) 5′- GTCAGGTCATCACTATCGGCAAT -3′	147
	(R) 5′- AGAGGTCTTTACGGATGTCAACGT -3′	
GPR109A	(F) 5′- GCTGCCCTGTCGGTTCAT -3′	134
	(R) 5′- CGTGGCTGACTTTCTCCTGAT -3′	
iNOS	(F) 5′- CACCCAGAAGAGTTACAGC -3′	186
	(R) 5′- GGAGGGAAGGGAGAATAG -3′	
COX-2	(F) 5′- AGAGTCAGTTAGTGGGTAGT -3′	170
	(R) 5′- CTTGTAGTAGGCTTAAACATAG -3′	
TNF-α	(F) 5′- CCACGCTCTTCTGTCTACTG -3′	145
	(R) 5′- GCTACGGGCTTGTCACTC -3′	
IL-1β	(F) 5′- TGTGATGTTCCCATTAGAC -3′	131
	(R) 5′- AATACCACTTGTTGGCTTA -3′	
IL-6	(F) 5′- AGCCACTGCCTTCCCTAC -3′	156
	(R) 5′- TTGCCATTGCACAACTCTT -3′	

### ELISA

The amounts of TNF-α, IL-1β, and IL-6 in the culture medium were measured with commercial ELISA kits obtained from BioLegend.

### Tyrosine hydroxylase and IBA-1 immunohistological analysis

The brains were fixed and processed for immunostaining as described previously [31]. The primary antibodies used in this study were as follows: rabbit polyclonal anti-tyrosine hydroxylase (TH) (1:1000; Abcam, Cambridge, CA, USA) and ionized calcium-binding adaptor molecule-1 (IBA-1) (1:200, Proteintech, Chicago, IL, USA). To determine cell numbers, total nigral TH-positive cells were counted by three researchers blind to the experimental design, and the average of these scores were reported.

### Western blot analysis

After the last behavioral test, the SNs of the rats were rapidly dissected out, frozen, and stored in a deep freezer at −80°C until the assays. The rat brain SNs and the microglial cells were lysed in lysis buffer (Beyotime Inst. Biotech, Beijing, China) according to the manufacturer’s instructions. Protein concentrations were measured using a bicinchoninic acid protein assay kit (Beyotime Inst. Biotech, Beijing, China). A total of 30 μg of protein was resolved by 10% SDS-polyacrylamide gel electrophoresis (SDS-PAGE) and transferred onto immunoblot polyvinylidene difluoride membranes (Millipore, Billerica, MA, USA). The blots were blocked with 5% nonfat milk in Tris-buffered saline with 0.1% Tween (TBS-T) for 1 h, washed three times with TBS-T, and incubated overnight at 4°C with primary antibodies against iNOS (1:2000), COX-2 (1:1000), OX-42 (1:1000), TH (1:1000) (Abcam, Cambridge, CA, USA), p-NF-κB p65 (1:1000) (Cell Signaling Technology, Danvers, MA, USA), GPR109A (1:300) and β-actin (1:2000) (Santa Cruz, CA, USA). The blots were then washed four times for 15 min each in TBS-T and incubated with a horseradish peroxidase-labeled secondary goat anti-rabbit (1:2000; Santa Cruz, CA, USA) or rabbit anti-goat antibody (1:2000; Santa Cruz, CA, USA) for 1 h at room temperature. Next, the blots were washed again four times for 15 min each in TBS-T. Membranes were visualized with enhanced chemiluminescence (ECL kit; Applygen Inst. Biotech, Beijing, China).

### Statistical analyses

The data are presented as the mean ± SD and were analyzed using SPSS 12.0 statistical software package (SPSS Inc., Chicago, IL, USA). The groups were compared by one-way analysis of variance (ANOVA) followed by the least significant difference test. A *P* value of less than 0.05 was considered statistically significant.

## Results

### Effect of β-hydroxybutyric acid on lipopolysaccharide-induced degeneration of dopaminergic neurons

**Figure 1:**
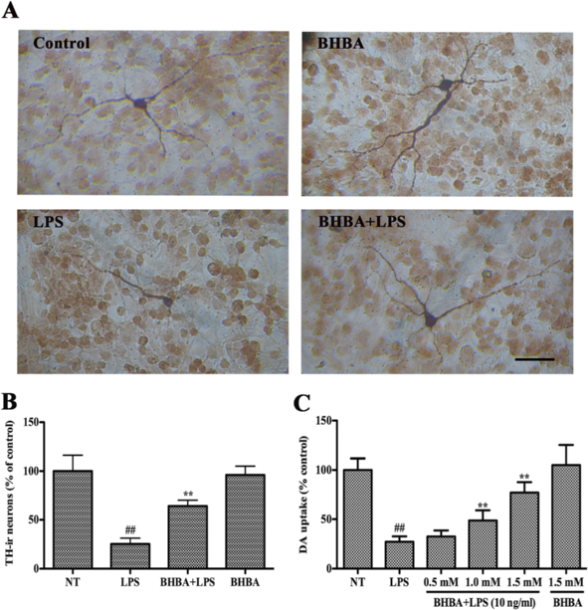
**Effects of β-hydroxybutyric acid (BHBA) on lipopolysaccharide (LPS)-induced degeneration of dopaminergic neurons in mesencephalic neuron-glia cultures.** Cultures were pretreated for 30 min with vehicle or indicated concentrations of BHBA before treatment with 10 ng/ml LPS. Seven days later, LPS-induced neurotoxicity was assessed by representative immunostaining images **(A)**, the TH-ir neuron count **(B)**, and the level of [^3^H]DA uptake **(C)**. The scale bar indicates 250 μm. The results are expressed as a percentage of the vehicle-treated control cultures and presented as the mean ± SD from three independent experiments performed in triplicate. **P <0.01 compared with the LPS-treated cultures; and ^##^ *P* <0.01 compared with the vehicle-treated cultures.

To investigate whether the LPS-induced damage of dopaminergic neurons could be prevented by BHBA, rat mesencephalic neuron-glia cultures were pretreated for 30 min with vehicle or BHBA (0, 0.5, 1, or 1.5 mM) before treatment with 10 ng/ml LPS. Seven days later, the degeneration of dopaminergic neurons was assessed by TH immunostaining and [^3^H]DA uptake. Morphologically, the remaining tyrosine hydroxylase-immunoreactive (TH-ir) neurons in the LPS-treated cultures had significantly fewer dendrites, and shorter or evenly truncated axons (Figure 1A). In the cultures pretreated with 1.5 mM BHBA before LPS stimulation, the TH-ir neurons were more numerous and appeared less affected compared with the LPS-treated cultures (Figure 1A). The LPS treatment reduced the number of TH-ir neurons by 75% (*P* <0.01) compared with the vehicle-treated control cultures (Figure 1B). BHBA (1.5 mM) significantly attenuated the LPS-induced loss of TH-ir neurons (Figure 1B). [^3^H]DA uptake assays showed that the LPS (10 ng/ml) treatment reduced the uptake capacity by approximately 70% of the vehicle control (*P* <0.01), and this LPS-induced reduction in [^3^H]DA was abated by pretreatment with BHBA in a concentration-dependent manner (Figure 1C). [^3^H]DA uptake in the cultures treated with 1.5 mM BHBA alone did not differ from that in the control cultures (vehicle-treated only), suggesting that BHBA is devoid of obvious toxicity (Figure 1C).

### β-hydroxybutyric acid administration improves functional recovery from lipopolysaccharide intranigral injection

**Figure 2:**
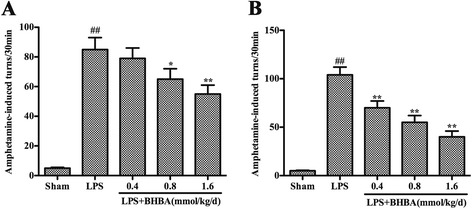
**β-hydroxybutyric acid (BHBA) treatment improves the behavioral dysfunction of lipopolysaccharide (LPS)-induced Parkinson’s disease (PD) model rats.** Rats were randomly grouped and then pretreated with BHBA (0.4, 0.8, or 1.6 mmol/kg/d) or vehicle 3 days before LPS injection and subsequently for 21 days after LPS injection (24 days in total). **(A, B)** The number of turns induced by apomorphine for the LPS-induced PD model rats after 2 and 4 weeks of BHBA administration. The results are expressed as the mean ± SD. *P <0.05 and **P <0.01 compared with the LPS-treated rats; and ^##^ *P* <0.01 compared with the sham-operated control rats.

A rotational behavior assay in animal models of PD can be used to characterize the extent of a lesion and/or to investigate the therapeutic effects of drug candidates. To determine the effect of BHBA treatment on motor dysfunction, LPS-induced PD model rats were subjected to behavioral tests at two and four weeks after LPS injection. Administration of amphetamine, which is an indirect agonist of DA receptor, elicits rotational behavior towards the injection side. The results of the rotational behavior assay showed that the BHBA treatment significantly attenuated amphetamine-induced rotation (Figure 2). These data indicated that the administration of BHBA had beneficial effects on motor dysfunction in the LPS-induced PD model rats.

### β-hydroxybutyric acid administration attenuates depletion of dopamine and 3,4-dihydroxyphenylacetic acid in the striatum induced by lipopolysaccharide intranigral injection

**Figure 3:**
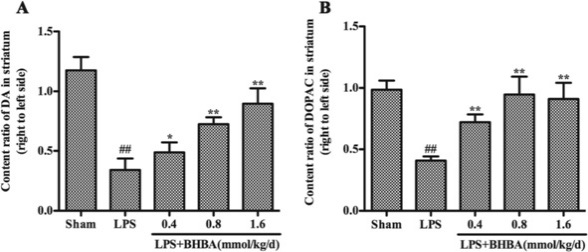
**Effects of β-hydroxybutyric acid (BHBA) treatment on the levels of dopamine (DA) and 3,4-dihydroxyphenylacetic acid** (**DOPAC) in the striatum.** Rats were randomly grouped and then pretreated with BHBA (0.4, 0.8, or 1.6 mmol/kg/d) or vehicle 3 days before lipopolysaccharide (LPS) injection and subsequently for 21 days after LPS injection (24 days in total). The levels of DA **(A)** and DOPAC **(B)** in the striatum were detected by high-performance liquid chromatography (HPLC), and the ratio of the right to left side was calculated. The results are expressed as the mean ± SD. *P <0.05 and **P <0.01 compared with the LPS-treated rats; and ^##^ *P* <0.01 compared with the sham-operated control rats.

The levels of DA and its metabolite DOPAC, in the rat brain striatum were measured by HPLC. As shown in Figure 3, in the vehicle-treated control group, the levels of DA and DOPAC on the LPS-injected side were reduced to 34% (*P* <0.01) and 41% (*P* <0.01) of the levels of the non-injected side, respectively. Treatment with BHBA (0.4, 0.8, or 1.6 mmol/kg/d) for 4 weeks significantly attenuated DA depletion in the striatum as induced by LPS intranigral injection (Figure 3A). The levels of DA on the LPS-injected side were 49% (*P* <0.05), 72% (*P* <0.01) and 90% (*P* <0.01) of the levels of the noninjected side in the animals treated with 0.4, 0.8, and 1.6 mmol/kg/d BHBA, respectively (Figure 3A). The levels of DOPAC on the LPS-injected side were 72% (*P* <0.01), 95% (*P* <0.01) and 91% (*P* <0.01) of the levels on the non-injected side in the groups treated with 0.4, 0.8, and 1.6 mmol/kg/d BHBA, respectively (Figure 3B).

### β-hydroxybutyric acid treatment increases the number of tyrosine hydroxylase (TH)-positive cells and TH expression in the substantia nigra of lipopolysaccharide-induced Parkinson’s disease model rats

**Figure 4:**
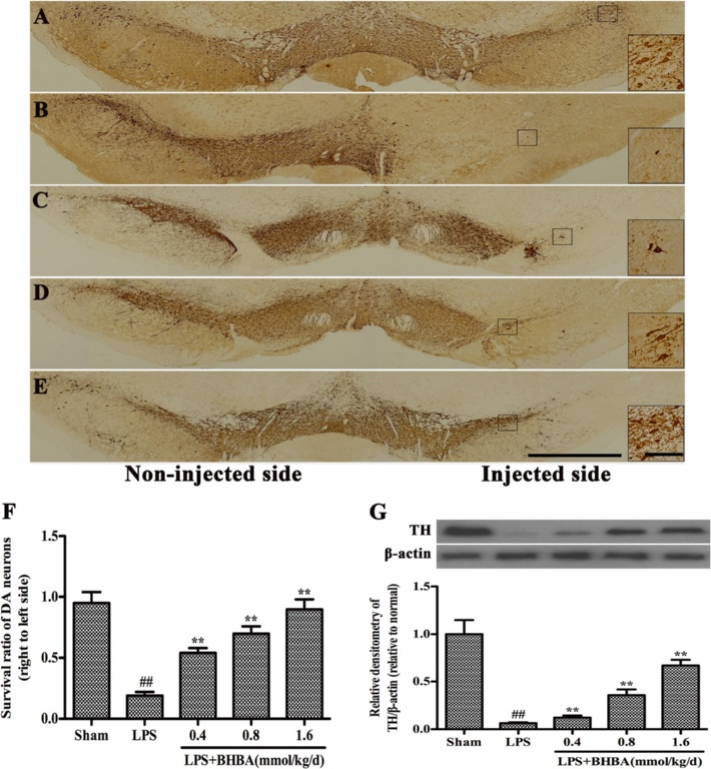
**β-hydroxybutyric acid (BHBA) treatment increases the number of tyrosine hydroxylase** **(TH)-positive cells and TH expression in the substantia nigra (SN) of lipopolysaccharide (LPS)-induced Parkinson’s disease (PD) model rats.** PBS or 10 μg LPS was unilaterally injected into the right SN of rats. The animals were sacrificed after 4 weeks of BHBA treatment. **(A-E)** Staining of TH-positive neurons in the SN. SN brain sections were processed for TH immunostaining. Insets are higher magnifications taken from the area outlined in lower magnification pictures. Scale bar of inset, 100 μm; scale bar of low magnification images, 1.0 mm. **(F)** The survival ratio of the dopaminergic neurons in the SNpc (the injected side versus the noninjected side) was calculated. **(G)** Western blot assay of TH expression. The experiments were repeated three times. A representative immunoblot is shown. The results are expressed as the mean ± SD. *P <0.05 and **P <0.01 compared with the LPS-treated rats; and ^##^ *P* <0.01 compared with the sham-operated control rats.

To further investigate the protective effect of BHBA on dopaminergic neurons, immunohistological analysis of TH expression was carried out in an *in vivo* study. In sham-operated animals, the numbers of TH-ir neurons were similar on the ipsilateral and contralateral sides to the injection site (Figure 4A). The survival rate of the TH-ir neurons was 95% (Figure 4F). The animals that received the vehicle treatment after LPS intranigral injection showed marked losses of TH-ir neurons and their dendrites (Figure 4B). Only 19% of the TH-ir neurons (*P* <0.01) in the SNpc on the LPS-injected side survived compared with those on the noninjected side (Figure 4F). In contrast, treatment with 0.4, 0.8, or 1.6 mmol/kg/d BHBA dramatically rescued this decline (Figure 4C-4F). TH, which is the rate-limiting enzyme in the synthesis of catecholamines, is critically involved in DA synthesis. We further investigated the expression of TH in the SN using western blot analysis. The results showed that the expression of TH significantly decreased in the SN of the LPS-induced PD model rats. BHBA administration markedly increased TH expression (Figure 4G), indicating that it rescued dopaminergic neuronal damage caused by LPS-induced neurotoxicity.

### β-hydroxybutyric acid treatment inhibits microglial activation induced by lipopolysaccharide intranigral injection

**Figure 5:**
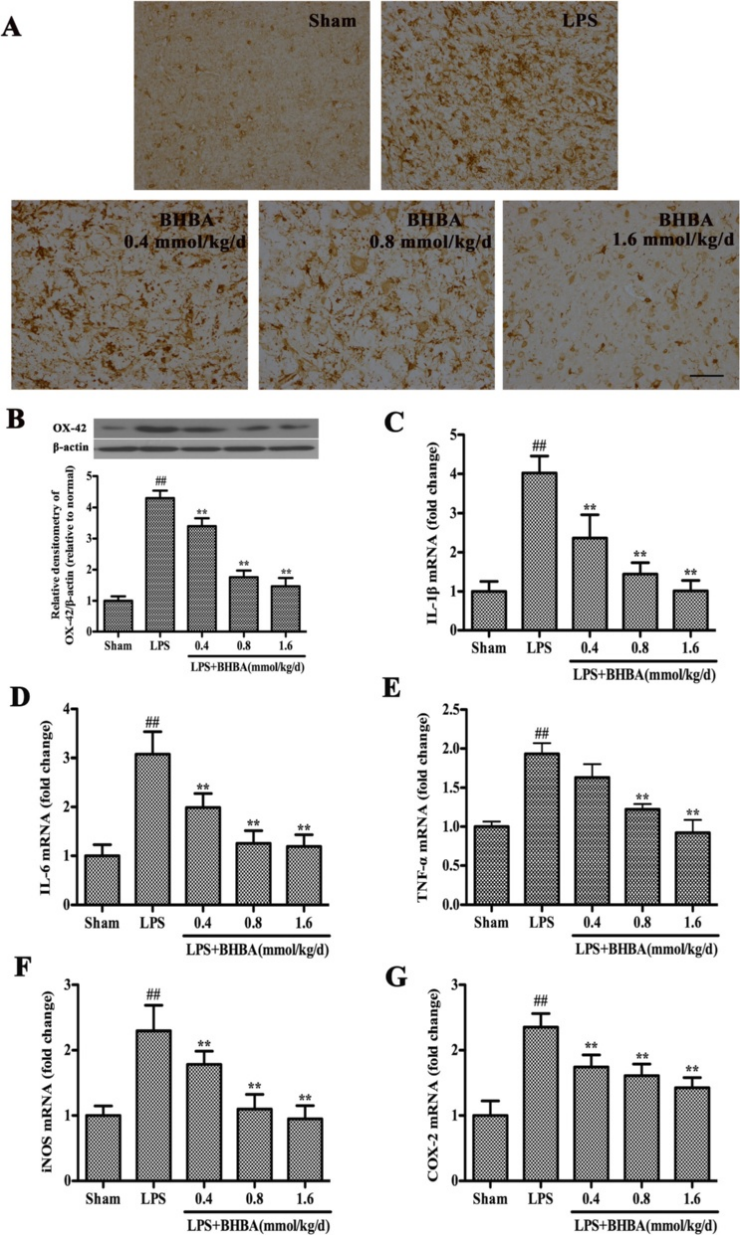
**β-hydroxybutyric acid (BHBA) treatment inhibits microglial activation and downregulates mRNA expression of pro-inflammatory mediators in the substantia nigra (SN) of lipopolysaccharide (LPS)-induced Parkinson’s disease (PD) model rats. (A)** The morphological changes of the microglia in the SN as shown by IBA-1 immunostaining. Representative photomicrographs of the SN area are shown. The scale bar indicates 100 μm. **(B)** Western blot assay of O-X42 expression. The experiments were repeated three times. A representative immunoblot is shown. **(C-G)** Real-time RT-PCR analysis of pro-inflammatory enzyme (iNOS and COX-2) and pro-inflammatory cytokine (TNF-α, IL-1β, and IL-6) expression in the SN of LPS-induced PD model rats. The data are expressed as fold changes relative to the sham-operated control rats. The results are expressed as the mean ± SD. *P <0.05 and **P <0.01 compared with the LPS-treated rats; and ^##^ *P <*0.01 compared with the sham-operated control rats.

To investigate whether the neuroprotective effect of BHBA is associated with the inhibition of LPS-induced microglial activation, we examined the expression of IBA-1, which is a specific marker for microglial activation. The activation of microglia was significantly suppressed by BHBA treatment in a dose-dependent manner (Figure 5A). To obtain quantitative data, the SN of the rats was dissected out, and microglial activation was determined by western blot analysis using an OX-42 antibody. The results confirmed that the BHBA treatment suppressed LPS-induced microglial activation (Figure 5B).

Because microglia are the main sources of pro-inflammatory enzymes and pro-inflammatory cytokines in the brain and BHBA inhibits microglial activation, we hypothesized that BHBA treatment could inhibit the LPS-induced expression of pro-inflammatory enzymes and pro-inflammatory cytokines. We measured the mRNA expression of iNOS, COX-2, TNF-α, IL-1β, and IL-6. As shown in Figure 5C-G, LPS injection significantly upregulated iNOS, COX-2, TNF-α, IL-1β, and IL-6 mRNA expression, and the BHBA treatment downregulated those expressions in a dose-dependent manner (Figure 5C-G).

### Lipopolysaccharide enhances expression of GPR109A in primary rat microglial cells

**Figure 6:**
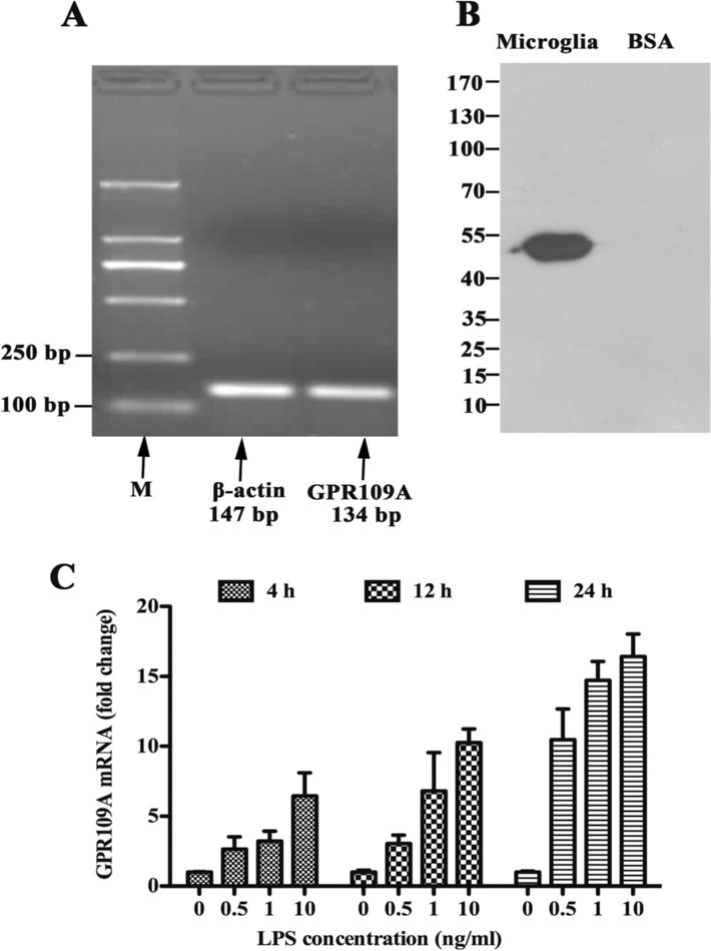
**Lipopolysaccharide (LPS) enhances expression of GPR109A in primary rat microglial cells. (A)** RT mixtures from primary rat microglial cells were carried out to detect GPR109A mRNA expression by PCR amplification (M, 2000 bp DNA marker). PCR products were visualized by 2% agarose gel electrophoresis, and the expected 134-bp GPR109A was detected in the primary rat microglial cells. **(B)** Western blot of GPR109A in primary rat microglial cells showing a specific band of the expected size at approximately 50 kDa. **(C)** Microglial cells were treated with 0, 0.5, 1, or 10 ng/ml of LPS for the indicated times. GPR109A mRNA expression was quantified by quantitative real-time RT-PCR and normalized to β-actin mRNA expression.

GPR109A is the functional receptor of BHBA, and its mRNA (Figure 6A) and protein (Figure 6B) were detected in primary rat microglial cells. To investigate whether a correlation exists between GPR109A expression and the degree of microglial activation, microglia were stimulated with LPS (0, 0.5, 1, or 10 ng/ml) for several time points. GPR109A mRNA expression was detected as early as 4 h after LPS stimulation and was observed to significantly increase in both dose- and time-dependent manners (Figure 6C). This finding revealed that GPR109A mRNA is expressed at low levels in unstimulated conditions and is induced in a time-dependent fashion in response to LPS, suggesting a role of GPR109A during the early stages of microglial activation.

### β-hydroxybutyric acid inhibits lipopolysaccharide-induced inflammation responses via GPR109A in primary rat microglial cells

**Figure 7:**
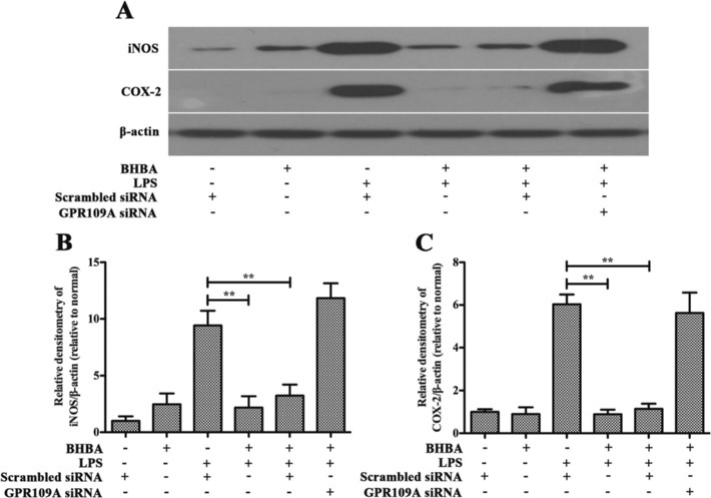
**β-hydroxybutyric acid (BHBA) inhibits lipopolysaccharide (LPS)-Induced production of pro-inflammatory enzymes via GPR109A in primary rat microglial cells.** Attenuation by BHBA (1.5 mM) of LPS-induced production of iNOS **(A, B)** and COX-2 **(A, C)** from primary rat microglial cells *in vitro*, this effect is abolished with silencing of GPR109A (**P <0.01).

**Figure 8:**
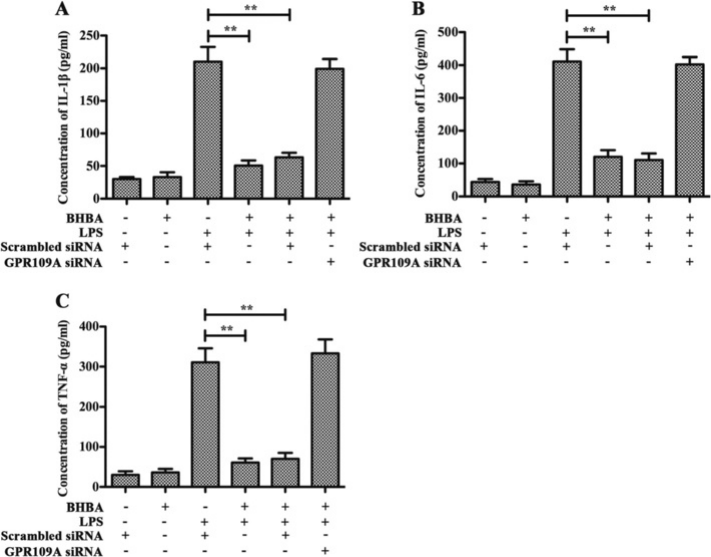
**β-hydroxybutyric acid (BHBA) inhibits lipopolysaccharide (LPS)-Induced release of pro-inflammatory cytokines via GPR109A in primary rat microglial cells.** Attenuation by BHBA (1.5 mM) of LPS-induced release of IL-1β **(A)**, IL-6 **(B)** and TNF-α **(C)** from primary rat microglial cells *in vitro*, this effect is abolished with silencing of GPR109A (A- C) (**P <0.01).

The incubations of non-siRNA-transfected, scrambled siRNA-transfected and GPR109A-siRNA transfected primary rat microglial cells were carried out in parallel. As shown in Figures 7 and 8, pretreatment with BHBA (1.5 mM) attenuated the LPS-induced increased production of iNOS, COX-2, TNF-α, IL-1β and IL-6 in both the nontransfected and scrambled siRNA-transfected cells, but in the cells with the knockdown of GPR109A by siRNA, this effect was abolished. These data suggest that BHBA inhibits the LPS-induced production of pro-inflammatory enzyme (iNOS and COX-2) and pro-inflammatory cytokine (TNF-α, IL-1β, and IL-6) through GPR109A.

### β-hydroxybutyric acid downregulates NF-κB activation via GPR109A

**Figure 9:**
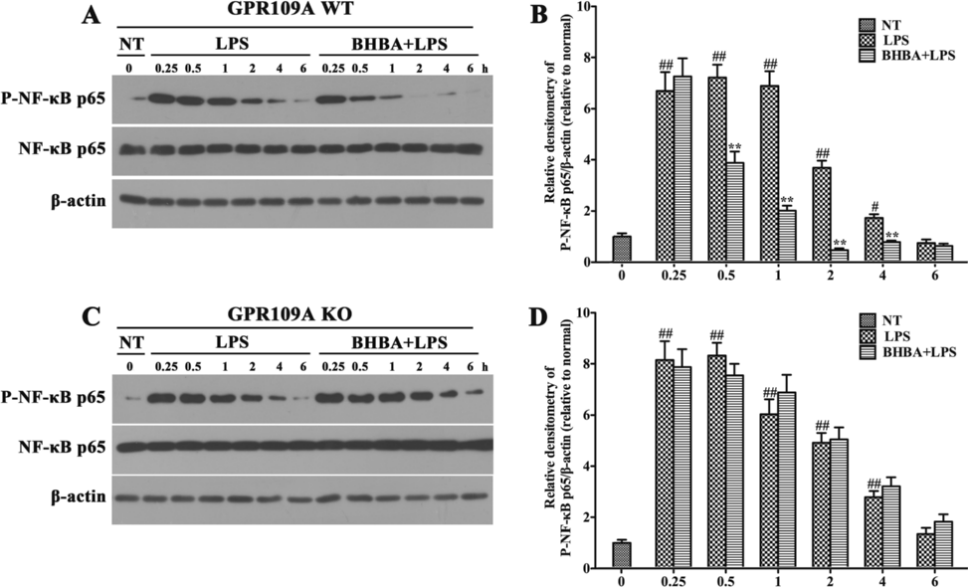
**β-hydroxybutyric acid (BHBA) downregulates NF-κB activation via GPR109A.** Primary rat microglial cells were treated with 0 or 10 ng/ml LPS for the indicated times in the presence or absence of 1.5 mM BHBA. Western blot was performed with the indicated antibodies. At 0.5, 1, 2, 4 h after lipopolysaccharide (LPS) stimulation, significant reductions in pNF-κB levels were observed in the BHBA-treated primary rat microglial cells (GPR109A WT) **(A, B).** In contrast, no difference was observed in pNF-κB levels between the vehicle- and BHBA-treated primary rat microglial cells with the silencing of GPR109A (GPR109A KO) **(C, D)**. Each immunoreactive band was digitized and expressed as a ratio of the β-actin level. The ratio of the control group band was set to 1.00. The data are expressed as the mean ± SD of three independent experiments. ^##^P <0.01 and ^#^P <0.05 indicated significant differences compared with the no-treatment group (NT). **P <0.01 indicated a significant difference compared with the BHBA-untreated LPS-stimulated group.

The NF-κB pathway is a key mediator of inflammation and is activated via toll-like receptors (TLRs), resulting in increased cytokine and chemokine production [32]. Moreover, transcription of iNOS, COX-2, TNF-α, IL- 1β, and IL-6 is regulated by the transcription factor NF-κB. To elucidate the inhibitory mechanism of BHBA on pro-inflammatory mediator production in primary rat microglial cells, we examined NF-κB signaling in response to LPS in primary rat microglial cells. Microglial cells were stimulated with LPS (10 ng/ml) for 0.25, 0.5, 1, 2, 4 and 6 h in the presence or absence of BHBA (1.5 mM). Cell lysates were subjected to western blotting for p-NF-κB p65, NF-κB p65 and β-actin. As shown in Figure 9, the level of active NF-κB p65 (p-NF-κB p65) peaked at 0.25 h after LPS stimulation. Levels of p-NF-κB p65 were maintained for 4 h; however, a striking reduction in its level was observed at 2 h after LPS stimulation (Figure 9A,B). As expected, BHBA significantly reduced its levels in primary rat microglial cells after LPS stimulation (Figure 9A,B). Knockdown of GPR109A with siRNA abolished this effect (Figure 9C,D).

## Discussion

Our findings demonstrated that BHBA exerted neuroprotective effects on dopaminergic neurons by inhibiting microglial activation in an *in vitro* model of LPS-induced dopaminergic neurodegeneration and an *in vivo* rat model induced by intranigral injection of LPS. The mechanistic study showed that the inhibitory effect of BHBA on microglia was mediated by GPR109A and involved the NF-κB signaling pathway, inhibiting pro-inflammatory enzyme (iNOS and COX-2) and pro-inflammatory cytokine (TNF-α, IL-1β, and IL-6) production. These data revealed that GPR109A-mediated signaling pathways might represent potential targets for therapeutic interventions to prevent or slow the progression of PD.

In recent years, the involvement of neuroinflammatory processes in the nigral degeneration of dopaminergic neurons in PD has gained increasing attention. In the CNS, microglia, which are the resident innate immune cells, play a major role in the inflammatory process. In addition, these cells have been found to be highly concentrated in the SNpc [18,33]. They are the resident macrophages of the brain and share similar properties [34,35] constituting 10% of brain cells [33]. Once activated, these microglia transform from striated bodies into large round, amoeboid, bodies with short, thick processes. In PD, activated microglia in the SNpc have been found to express pro-inflammatory enzyme (iNOS and COX-2) and pro-inflammatory cytokine (TNF-α, IL-1β, and IL-6) [17,36]. Most evidence has indicated that pro-inflammatory enzymes and pro-inflammatory cytokines may mediate neuronal degeneration [37-39].

LPS, which is an endotoxin from Gram-negative bacteria, is a potent stimulator of microglia, and *in vivo* and *in vitro* PD models induced by LPS are widely used to study the inflammatory process in the pathogenesis of PD. These PD models have also been widely used in drug discovery, and a variety of agents have been evaluated for their potential neuroprotective effects in LPS-induced PD models, such as FLZ, triptolide, and urocortin [21,22,40]. In a mesencephalic mixed neuron-glial culture, LPS have been shown to induce microglial activation, and activated microglia have been demonstrated to release the proinflammatory and cytotoxic factors NO, TNF-α, and IL- 1β, leading to the consequent degeneration of dopaminergic neurons [20]. LPS injected into the SN of rats induce microglial activation and dopaminergic neuron loss [41]. Moreover, there is no detectable damage to either GABAergic or serotoninergic neurons in the striatum and nigra after LPS injection, indicating that LPS selectively induce dopaminergic neuron death in the nigrostriatal system [42]. More recent studies have confirmed these results, also finding increased levels of proinflammatory mediators, including IL-1β, TNF-α, IL-6, and NO, in the SN after LPS injection, which may be causal factor of LPS-induced neuronal damage [21,43,44]. In addition, the effects of intranigral LPS injection on behavior and DA content and turnover have been investigated, and it has been shown that LPS treatment enhances locomotor activity two- to threefold and increases DA turnover ratios in comparison with control subjects. These findings suggest that LPS insult may induce a compensatory response of the dopaminergic system [22]. Therefore, *in vitro* and *in vivo* LPS PD models represent powerful tools for mechanistic studies and the identification of potential therapeutic agents.

BHBA is an important intermediate of amino and fatty acid catabolism that has been reported to be effective in the treatment of a variety of inflammatory and autoimmune diseases, such as colonic inflammation and experimental allergic encephalomyelitis (EAE) [45,46]. A previous study has reported that BHBA has potent neuroprotective effects on dopaminergic neurons both *in vitro* and *in vivo*. Yoshihiro *et al.* have found that BHBA protects cultured mesencephalic neurons from MPP^+^ toxicity and hippocampal neurons from Aβ_1–42_ toxicity [24]. *In vivo* administration of BHBA confers partial protection against dopaminergic neurodegeneration and motor deficits induced by MPTP, and these effects appear to be mediated by a complex II-dependent mechanism that leads to improved mitochondrial respiration and ATP production [25]. Soyeon *et al.* have proven that BHBA extends the life span, attenuates motor deficits, and prevents striatal histone deacetylation in transgenic R6/2 mice [47]. To elucidate whether its neuroprotective activity involves an anti-inflammatory function, we investigated the effect of BHBA on LPS-induced damage to dopaminergic neurons in a primary mesencephalic neuron/glia mixed culture. We found that BHBA concentration-dependently attenuated the LPS-induced decrease in [^3^H]DA uptake and loss of TH-ir neurons in a primary mesencephalic neuron/glia mixed culture. In the current *in vivo* study, we investigated the motor dysfunction of these PD model rats using a rotational behavior assay. Because LPS was injected on one side of the SN, apomorphine-induced rotation to the lesioned side was used to evaluate the degree of damage to the dopaminergic system. Apomorphine-induced rotation significantly increased in the LPS-induced PD model rats, and BHBA showed therapeutic effects on this behavioral dysfunction. Further experiments demonstrated that BHBA inhibited LPS-induced microglial overactivation, pro-inflammatory factor release and dopaminergic neuronal damage. These data suggest that BHBA plays a neuroprotective role through inhibiting microglial overactivation.

GPR109A (PUMA-G in mice and HM74A in humans) is a seven-transmembrane G-protein-coupled receptor of the Gi family that is expressed mainly in white adipocytes and immune cells, such as monocytes and neutrophils [47]. BHBA has been identified as an endogenous ligand of GPR109A [48]. The anti-inflammatory effects of BHBA are mediated by the activation of GPR109A [47]. Accumulating data have demonstrated a strong anti-inflammatory activity of BHBA in macrophages, monocytes, adipocytes, and retinal pigment epithelial cells. *In vitro* experiments have demonstrated that BHBA inhibits pro-inflammatory cytokine production, LDL uptake, and chemotaxis in macrophages via activating GPR109A [49]. Moreover, BHBA inhibits the expression of TNF-α, IL-6 and MCP-1 in human monocytes stimulated by LPS [50]. *In vivo* experiments have shown that GPR109A mediates the therapeutic effects of DMF in EAE [46]. In this study, we found that the level of GPR109A expression was correlated with the degree of microglial activation, as measured by proinflammatory cytokine production. Therefore, we hypothesized that activated microglia may be subjected to negative feedback mechanisms via GPR109A signaling.

We further assessed the mechanism underlying the anti-inflammatory effect of BHBA in primary rat microglial cells and found that it significantly inhibited LPS-induced proinflammatory mediator production. The knockdown of GPR109A with siRNA resulted in the loss of this anti-inflammatory effect in primary rat microglial cells. Because NF-κB is clearly one of the most important regulators of pro-inflammatory gene expression [51], we examined whether GPR109A-mediated signaling pathways modulate NF-κB signaling and found that BHBA inhibits pro-inflammatory cytokines via NF-κB inactivation in primary rat microglial cells. Furthermore, we demonstrated that the inhibitory effect of BHBA is mediated by GPR109A.

## Conclusions

In conclusion, this study demonstrates that BHBA treatment improves LPS-induced behavioral dysfunction and protects dopaminergic neurons through inhibiting microglia-mediated neuroinflammation both *in vitro* and *in vivo*. Several lines of evidence presented in this study demonstrate that BHBA provides potent neuroprotection to dopaminergic neurons against LPS-induced neurotoxicity through the regulation of GPR109A-mediated signaling pathways. Thus, GPR109A-mediated signaling pathways may represent potential targets for therapeutic intervention to prevent or slow the progression of PD.

## Abbreviations

BHBA: β-hydroxybutyric acid
COX-2: Cyclooxygenase-2
DA: Dopamine
DMEM: Dulbecco’s modified Eagle medium
FBS: Fetal bovine serum
GPR109A: G-protein-coupled receptor 109A
IBA-1: Ionized calcium binding adaptor molecule-1
IL-6: Interleukin 6
iNOS: Inducible nitric oxide synthase
LPS: Lipopolysaccharide
L-1β: Interleukin 1β
NF-κB: Nuclear factor κB
PBS: Phosphate-buffered saline
PD: Parkinson’s disease
SN: Substantia nigra
TH: Tyrosine hydroxylase
TNF-α: Tumor necrosis factor alpha
